# Plague Inoculation

**Published:** 1902-12-20

**Authors:** 


### PLAGUE INOCULATION.

J. H. THORNTON, C.B., M.B., B.A., Deputy Surgeon-General I.M.S. (retired), writes: A statement has appeared in the *Times* to the effect that the anti-plague inoculations in the Punjab have been suspended in consequence of many deaths having occurred from them. My recent warning against these inoculations, addressed to the Secretary of State for India, has thus been fully justified, and it is to be hoped that these empirical and dangerous operations will now be countermanded, and pseudo-scientists prevented in future from making their reckless and unjustifiable experiments upon the helpless population of India.

